# Prevalence, Clinical Signs, Diagnosis and Treatment of Post-Pandemic SARS-CoV-2 Infection in Cats in 2023: Co-Infection with FHV, FCV, *Mycoplasma* spp. and *Chlamydia felis*—A Single-Center Study in Bulgaria

**DOI:** 10.3390/vetsci13040374

**Published:** 2026-04-13

**Authors:** Ivo Sirakov, Milena Krastanova, Nikolina Rusenova, Stoyan Shishkov, Anton Rusenov, Bilyana Sirakova, Kalina Mihova, Kalina Shishkova

**Affiliations:** 1 Department of Medical Microbiology, Faculty of Medicine, Medical University of Sofia, 2 Zdrave Str., 1431 Sofia, Bulgaria; 2 Department of Anatomy, Physiology and Animal Sciences, Faculty of Veterinary Medicine, University of Forestry, 1756 Sofia, Bulgaria; milenakrastanova777@gmail.com; 3 Department of Veterinary Microbiology, Infectious and Parasitic Diseases, Faculty of Veterinary Medicine, Trakia University, 6000 Stara Zagora, Bulgaria; n_v_n_v@abv.bg; 4 Laboratory of Virology, Faculty of Biology, University of Sofia, 8 Dragan Tzankov Blvd., 1164 Sofia, Bulgaria; sashishkov@yahoo.com (S.S.); biborisova07@gmail.com (B.S.); k_shishkova@biofac.uni-sofia.bg (K.S.); 5 Department of Internal Diseases, Faculty of Veterinary Medicine, Trakia University, 6000 Stara Zagora, Bulgaria; vetroussenov@abv.bg; 6 Molecular Medicine Center, Department of Medical Chemistry and Biochemistry, Faculty of Medicine, Medical University of Sofia, 1431 Sofia, Bulgaria; kalina_mihova@abv.bg

**Keywords:** SARS-CoV-2, post-pandemic, animals, co-infection, upper respiratory tract, pathogens

## Abstract

SARS-CoV-2 is a zoonotic virus with a proven ability to infect various animal species, including domestic cats. In the post-pandemic period of COVID 19, limited data still exist on the clinical course, shedding of infectious virus and diagnostic features in cats. The aim of this study was to investigate the spread of SARS-CoV-2 in cats in 2023, the clinical manifestations of the infection, the diagnostic algorithm, including molecular detection of viral components, the differential diagnosis of co-infection with FHV, FCV, *Mycoplasma* spp. and *Chlamydia felis*, serology, and the isolation of infectious SARS-CoV-2. The immunomodulatory therapy in animals with a standalone SARS-CoV-2 infection was applied. The study included oropharyngeal, conjunctival and nasal swab samples from 102 domestic cats with clinical signs. Of them, 20.6% (21/102) were positive for SARS-CoV-2, with 16.67% (17/102) of the cats showing various variants of co-infection with FHV, FCV, *Mycoplasma* spp. and *Chlamydia felis*. Four of the cats had a standalone SARS-CoV-2 with mild clinical manifestations that included serous discharges from the eyes, without change in the general condition. The virus was isolated from these samples. These four cats and their owners were positive for antibodies to the virus, and the owners were PCR-negative. The treatment of SARS-CoV-2 infection included the preparations Viusid, RX immunosuport, Vetomun and Lisymun. This is one of the first post-pandemic studies covering FHV, FCV, *Mycoplasma* spp. and *Chlamydia felis* in domestic cats with SARS-CoV-2 infection and further expands on the essential main idea including the specified pathogens of interest.

## Simple Summary

The SARS-CoV-2 pandemic, COVID-19, has primarily affected humans, but has also affected domestic cats. Cats can transmit the virus through secretions from the mouth, nose, throat and feces without clinical symptoms and signs of disease. The study included 102 cats in Bulgaria in 2023 that showed signs of disease. The frequency of SARS-CoV-2 infection and its impact on animals was monitored. The tests we used included virus detection, virus cultivation in cell lines, antibody testing and screening for other pathogens such as *Feline herpesvirus, Feline calicivirus, Chlamydia felis* and *Mycoplasma* spp. In 21 cats, the SARS-CoV-2 test was positive and only four of the cats were free of infection with other pathogens. Most of the cats studied are domestic, with currently healthy owners, suggesting probable transmission of the infection from people who were asymptomatic or had recovered. Clinical signs in the cats were mild—discharge from the eyes, sneezing and lethargy. The animals recovered after supportive treatment to stimulate the immune system. SARS-CoV-2 was isolated from the four cats, which were not carriers of the other pathogens studied. Successful cultivation shows that the virus was infectious and it can be concluded that domestic cats can become infected and spread SARS-CoV-2, even with mild symptoms. Understanding the circulation of the virus among cats helps to prevent animal and human health.

## 1. Introduction

SARS-CoV-2 caused the pandemic in 2019 and, in addition to humans, it causes active infection in various animal species such as ferrets [1], minks, dogs and cats [2,3]. Among domestic animals, cats are more susceptible [2,4]. They shed the virus from the oropharynx and feces [5], which can be detected by Real Time PCR [5,6,7,8,9] and conventional PCR [6,10]. Isolation and cultivation of the virus in permissive cell cultures, such as VERO E6, can demonstrate its infectivity [11,12]. However, some SARS-CoV-2 PCR-positive samples from cats result in unsuccessful viral isolation [13]. Successful isolation of infectious virus from PCR-positive feline samples remains challenging, given that its isolation requires samples to be free of other intracellular pathogens, suggesting limitations in current diagnostic approaches. Other approaches for detection of infection in cats include serological assays [8,14,15,16,17,18]. They are suitable for retrospective studies to provide additional information on the viral spread during a period of interest. Infection can be asymptomatic [19] or the clinical picture may include mild to moderate upper respiratory tract (URT) symptoms such as sneezing and coughing [13,20], in addition to gastrointestinal symptoms [21]. In some cases, there can be complications, such as hypertrophic cardiomyopathy [22], myocarditis, pneumonia [23], and bronchial pneumonia [19]. Serological studies have further contributed to the understanding of SARS-CoV-2 exposure in cats, especially in retrospective analyses. However, reported seroprevalence varies considerably depending on the population studied.

Studies have shown varying data on the number of SARS-CoV-2 seropositive cats during the pandemic. Depending on whether the cats are of unknown status or live with SARS-CoV-2-positive owners, reports range from 0 to 22.47% [15,16,17] to 43% [24]—47.5% [8], respectively. In stray cats, these values can range from 0.8% [25] to 83% [18].

Detected cases of SARS-CoV-2 infection in domestic cats using molecular assays vary from 0.25—0.38% [15,26] to 11.25% [8]. Other studies have used antigen tests to detect SARS-CoV-2, reporting 23.60% positive cases in stray cats [27]. These discrepancies highlight significant heterogeneity in study design, sampling strategies, and diagnostic methodologies.

Domestic cats that have no outdoor access acquire the infection from their owners [8,28]. Data on the number of infected cats with outdoor access depends on the degree of compliance with anti-epidemic measures in the respective countries and regions, as the movement of people and their cats contributes to the spread of the virus in the environment [18,29].

There is a growing body of data on SARS-CoV-2 infection in cats. However, most studies have focused on serological prevalence or molecular detection of viral RNA, with less detailed attention given to their correlation with the clinical course of infection under natural conditions. There are still limited data on indoor cats with clinical manifestations and probable infection due to contact with asymptomatic or oligosymptomatic owners, especially in the context of complex diagnostic evaluation. In addition to the variability in detection rates, there are also gaps in the recognition of the clinical significance of SARS-CoV-2 infection in cats. Although infection is often asymptomatic, mild to moderate signs of upper respiratory and gastrointestinal disease may occur. More severe complications such as myocarditis, pneumonia, or bronchopneumonia have also been reported by author teams. Despite all the reported facts, the relationship between virus detection, shedding of infectious virions, and clinical manifestations under natural conditions remains poorly characterized. To a large extent, existing studies have focused either on serological tests or on molecular detection of genomic RNA, without including additional diagnostic approaches. Regarding clinically affected domestic cats that are presumed to have been infected by their owners, especially in the context of co-infections with other common feline respiratory pathogens, such as *Feline herpesvirus* (FHV), *Feline calicivirus* (FCV), *Mycoplasma* spp. and *Chlamydia felis*, existing data are scarce. In addition, there is a lack of information on studies evaluating therapeutic approaches as well as the importance of immunomodulatory treatment in infected animals. Reports have detected active SARS-CoV-2 infection in cats [8,10,26,27] and have demonstrated the possibility for virus circulation among cats and through environmental routes [14,18]. Hence, there are recommendations for the inclusion of SARS-CoV-2 in the diagnostic panel along with *Feline herpesvirus* (FHV), *Feline calicivirus* (FCV), *Mycoplasma* spp. and *Chlamydia felis* [10,30].

The aim of this study was to investigate the prevalence of post-pandemic SARS-CoV-2 in cats in 2023 and the clinical signs of infection. In our study, we associate the post-pandemic period—as declared by the WHO—with a distinctly different scientific, epidemiological and immunological context in the population. This phase is associated with both a change in the circulating variants of SARS-CoV-2 and in the severity of the infection. Logically, the question also arises about the infectivity of the virus, which could be proven in vitro. In this post-pandemic period, according to the WHO, we are talking more about a late phase of SARS-CoV-2 circulation from a biological, epidemiological and immunological point of view. The described changes could affect the dynamics of interspecies circulation of the virus, including in the cat population. The diagnostic algorithm included molecular detection, viral isolation in cell culture, serological testing and co-infections with FHV, FCV, *Mycoplasma* spp. and *Chlamydia felis*. Additionally, we evaluated the effect of immunomodulatory therapy in animals infected only with SARS-CoV-2.

## 2. Materials and Methods

### 2.1. Inclusion Criteria and Sampling

In line with our previous recommendation [10], the Bayvet Veterinary Clinic in Sofia, Bulgaria, included SARS-CoV-2 in the diagnostic panel for infections of the upper respiratory tract and eyes in cats caused by FHV, FCV, *Mycoplasma* spp. and *Chl. felis*. Hence, the inclusion criteria were the presence of symptoms such as nasal discharge, conjunctivitis, redness in the oral cavity (gingivitis, stomatitis, pharyngitis and other pathologies), gastrointestinal (GI) disorders (diarrhea) of unknown etiology, as well as the absence of indications that the diet was the cause of the GI disorder.

Oropharyngeal, conjunctival and nasal samples were collected with nasopharyngeal polypropylene swabs, CE (Llins Service & Consulting GmbH, Heidelberg, Germany). The swabs were placed in 1.5 or 2.0 mL tubes with 450 µL of saline. The samples were collected according to clinical manifestations and placed in a common tube. Material for nucleic acid extraction was taken from this tube.

Storage and transport of fecal samples or swab samples was done in 2.0 mL tubes containing 450 µL of physiological saline.

To obtain the virus in cell culture, the samples from cats must be negative for other agents that cause similar symptoms, namely FHV, FCV, mycoplasma and chlamydia (to prevent contamination of the cell culture).

The study included 102 cats from 5 January to 17 December 2023. The owners provided the medical history.

The cats that met the eligibility criteria for virus isolation underwent a physical examination—measurement of body temperature, pulse and respiration. Blood samples were taken for a complete blood count (CBC) and swab samples were taken for PCR analysis.

The owners whose cats met the criteria also had nasal and oropharyngeal swab samples taken for PCR detection of SARS-CoV-2.

All owners completed informed consent forms for the study.

The owners whose cats tested positive for SARS-CoV-2 were surveyed on:

1. How the cats were kept: indoors only, indoors with outdoor access or outdoors only;
2. Whether the cat had been in contact with other cats in the last 7–10 days;
3. Whether any family member had COVID-19 symptoms at the time, or when a family member had last been ill.

The owners filled out informed consent forms for the study, but did not give permission for subsequent blood samples to be taken from their cats (20 days later) for testing for antibodies against SARS-CoV-2, as the cats were aggressive, and the owners wished to avoid further stress to their pets.

### 2.2. Detection of Antibodies Against SARS-CoV-2

Blood samples for detection of antibodies against SARS-CoV-2 were collected from the ring finger of the owners whose cats met the criteria for virus isolation. Blood samples were collected using BD Microtainer^®^ contact-activated lancets (Becton Dickinson Holdings Pte Ltd., The Strategy, Singapore). Immunochromatographic lateral flow assays detected anti-SARS-CoV-2 IgG and IgM separately; Artron Rapid COVID-19 Antibody Test ^CE^ (Artron, BC, Canada) according to the manufacturer’s instructions was used.

Serum was separated from the blood samples obtained from the cats for CBC and was tested for the presence of antibodies against SARS-CoV-2 using ID SCREEN^®^ SARS-CoV-2 Double Antigen multi species ELISA (IDvet, 34790 Grabels, France). To remove non-specific viral inhibitors (thermolabile), the serum samples were heated to 56 °C for 30 min prior to testing.

### 2.3. Nucleic Acids Extraction and PCR

To obtain DNA/RNA from clinical samples and cell cultures, we used a viral RNA/DNA extraction kit (Jena Bioscience, Jena, Germany) according to the manufacturer’s instructions. To detect SARS-CoV-2, we used a two-step reaction, performing reverse transcription to obtain cDNA using the SCRIPT cDNA Synthesis Kit (Jena Bioscience, Jena, Germany) in a volume of 20 µL as follows: RNA 4 µL, dNTP 1 µL, reverse transcriptase 0.5 µL, RNase inhibitor 0.5 µL, SCRIPT RT buffer 4 µL, PCR water 8 µL and 2 µL of external primers: Ext. 2019nCorV F 5′-GGCAGTAACCAGAATGGAGA-3′ (positions 28346–28365) and Ext.2019nCorV R 5′-CTCAGTTGCAACCCATATGAT-3′ (positions 28681–28661), forming a 335 bp fragment. The reaction parameters were as follows: 42 °C for 10 min; 50 °C for 30 min and 70 °C for 10 min. After obtaining DNA copies, PCR was performed with 2 µL of internal primers: intF (5′-CACCGCTCTCACTCAACAT-3′), position 28432–28450 and intR (5′-CATAGGGAAGTCCAGCTTCT-3′), position 28643–28624, PCR water 6 µL, 2 µL of cDNA and 10 µL of Hot Start Ruby MasterMix 2× (Jena Bioscience, Jena, Germany) and the following protocol: 30 cycles of denaturation at 95 °C for 10 s, annealing at 54.6 °C for 20 s and elongation at 72 °C for 30 s; final extension at 72 °C for 10 min and storage at 10 °C. Product size: 212 bp.

We also used the extracted DNA/RNA and the resulting cDNA to perform a differential diagnosis to detect FHV, *Chlamydia felis*, FCV and *Mycoplasma* spp. by PCR [10]. Since the variety of mycoplasma species that infect cats has not yet been fully characterized [31,32], we used primers for *Mycoplasma* spp.

We used PCR water as a negative control for PCR reactions.

### 2.4. Control of the Obtained Nucleic Acids and PCR Products

To control the obtained nucleic acids and PCR products, a NanoDrop 2000 (Thermo Fisher Scientific, Waltham, MA, USA) and gel electrophoresis were used. Gel electrophoresis was performed with 2% agarose (Lonza, Allendale, NJ, USA), 10 ng/mL ethidium bromide (Sigma, USA), 100 mL 1 × TAE buffer and a 100 bp DNA marker (Bioline, Meridian, MS, USA) at 130 V, 80 mA for 30 min. The result was observed on a UV transilluminator with a wavelength of 240/260 nm.

### 2.5. Cultivation, Isolation and Detection of SARS-CoV-2 from Cats

The samples were centrifuged at 3000 rpm for 20 min, and the supernatant was filtered through a 0.22 µm pore size filter and used for virus cultivation in the VERO E6 cell line, gene clone 76 (obtained from ATCC CRL-1587). The cells were maintained in Dulbecco’s Modified Eagle Medium (DMEM) (Sigma-Aldrich, Burlington, MA, USA, Merck, Darmstadt, Germany) low-glucose medium, 20 mM Hepes buffer (Sigma-Aldrich, Merck, Germany). Ten percent fetal calf serum (FCS) (Sigma) was added for cell culture and 5% was added for maintenance medium. Viruses were propagated in a 24 h monolayer culture with DMEM maintenance medium (with 4% FBS). For primary isolation, the infected cell cultures were monitored until day 5 from inoculation. Monolayers without cytopathic changes underwent up to 3 consecutive blind passages. For a culture result to be considered positive, the virus must undergo three successful passages with the appearance of a specific cytopathic effect in each of them. Successful passage through three consecutive passages is evidence of the presence of viable virus capable of infecting cells. Viral titer was determined using the Reed and Muench method [33]. Cultured human SARS-CoV-2 served as a positive control for the virus isolated from cats. From the obtained isolates, we extracted RNA using the ISOLATE II RNA Mini kit (Bioline, Meridian Bioscience, Memphis, TN, USA) and performed PCR to detect SARS-CoV-2 using the method described above. We used the PCR products obtained to perform sequence analysis.

### 2.6. Sequencing

The procedure was performed as previously described [34]. Enzymatic purification was performed according to the manufacturer’s instructions (Exo-CIP Rapid PCR Cleanup Kit, New England Biolabs, Ipswich, MA, USA) to degrade the residual primers and nucleotides in 1.5 μL aliquots of amplified samples from cell culture. Sequencing was carried out using an internal forward and internal reverse primer (Big Dye Terminator kit, v3.1, Applied Biosystems, Bedford, MA, USA), following the manufacturer’s instructions. To remove residual labeled nucleotides and primers, we used EDTA/sodium citrate/ethanol precipitation. Sequencing results were read on an automated capillary sequencing instrument (ABI 3500xl, Applied Biosystems, Bedford, MA, USA).

The obtained sequences were processed and identified using MEGA X [35] and BLAST NCBI (National Center for Biotechnology Information, Bethesda, MD, USA, https://blast.ncbi.nlm.nih.gov/Blast.cgi, accessed on 2 January 2026), respectively.

### 2.7. Statistics/Data Analysis

Demographic analysis included the following: sex and age distribution; positives for SARS-CoV-2 only; negatives for SARS only; negatives in all assays; mixed SARS-CoV-2 infections with one, two or more other causative agents; and distribution of other infections with and without SARS.

## 3. Results

We performed demographic analysis of the cats tested for SARS-CoV-2 between 5 January and 17 December 2023 (Table 1A,B), as age data were available for 71 of 102 cats

The study included a total of 102 samples from symptomatic cats meeting the inclusion criteria: redness in the oral cavity, nasal discharge and eye discharge. The fecal samples were not tested due to the lack of patients that met the eligibility criteria.

The PCR results showed that 31 out of the 102 cats, or 30.39%, tested negative for all pathogens: 12 females and 19 males with an average age of 2 years (min. 0.5–max. 9). There were 20 positive samples for FHV (19.6%), 17 for *Chl. felis* (16.7%) and 61 for mycoplasma (59.8%). The cats that were positive for SARS-CoV-2 were 21 or 20.6% (15 females and 6 males) (Table 2A,B). Differential diagnosis identified 17 SARS-CoV-2-positive cases (80.95%) with a mixed infection with some of the other disease-causing agents (Table 2). We also detected co-infections without SARS-CoV-2 involvement—16 cases (Table 3), with a total of 33 cases (46.49%) of co-infection among all 71 positive cases. FCV was not detected in the 102 samples tested here. Only 19% of the SARS-CoV-2 positive samples (from 1 male and 3 female cats) met the criteria for virus isolation and had clinical manifestations of standalone SARS-CoV-2 infection.

Our survey of the owners of the 21 SARS-CoV-2-positive cats showed that 14 of the cats were kept indoors and had no contact with other cats, four had outdoor access, and three owners did not respond. None of the owners or members of their families had had a current confirmed SARS-CoV-2 infection. Five owners reported a mild respiratory illness in the family with no tests performed, about 17–20 days before illness onset in the cats. Off the record, all owners reported being immune after the pandemic as a result of vaccination or previous illness.

**Figure 1:**
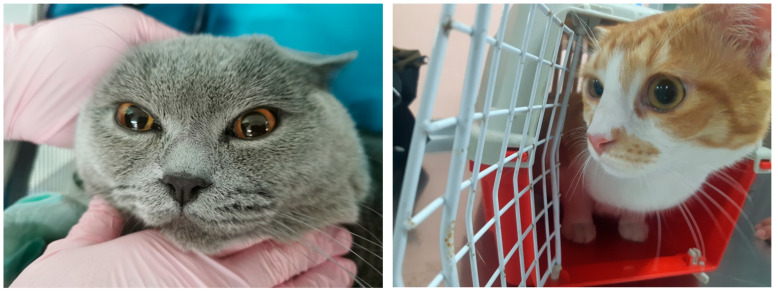
Serous discharge from the medial eye corner of both eyes of Cat 2 (**left**) and serous discharge from the left medial eye corner of Cat 3 (**right**).

History and clinical presentation of cats with standalone SARS2: On 8 April 2023, the owners of Cat 1 (reg. no. 270/080423), a 2.5-year-old female weighing 3 kg, visited a veterinary clinic because of serous discharge from the eyes. Cat 2 (reg. No. 271/110423), male, 1 year old, 5 kg, was admitted to the clinic on 11 April 2023 for castration when examination revealed slight serous discharge from the eyes. On 20 April 2023, the owner of female Cat 3 (reg. no. 276/200423), aged 10 months, weighing 4 kg, visited the same clinic due to serous discharge from the eyes (Figure 1). On 27 September another patient (Cat 4), a 3-year-old female, also with discharge from the eyes, was admitted to the clinic.

Medical history reported by the owners showed that the cats were kept indoors, i.e., they had no contact with the outdoor environment; there were no family members with COVID-19 symptoms; the owners of two of the cats had received three doses of vaccine (last dose in 2022); and the owner of the third cat had recovered from the disease in 2022 with an antibody titer of 655 BAU/mL (detected on 8 March 2023, doc. No. 8390202). The owners of Cat 4 did not provide information about their immune status.

The clinical examinations of the four cats showed no changes in their general condition. The body temperature, pulse, and respiration were within reference values. There was only serous secretion from the medial eye corner (Figure 1).

Analysis of blood parameters in all four cats showed values within the normal ranges: WBC from 2.9 to 8.8 × 10^9^/L, Lymph. 1.1–4.7 × 10^9^/L, Mon. 0.2–0.3 × 10^9^/L, Gran. 1.6–3.8 × 10^9^/L, HGB 107–153 g/L, HCT 33.7–38.4% and Eos. 21.3–29.8%. Erythrocytes (RBC) ranged from 6.92 × 10^12^/L to 9.52 × 10^12^/L.

The swab samples from the cats tested positive for SARS-CoV-2 by PCR while those from the owners tested negative. Serological samples from the cats taken during the illness were positive for antibodies against SARS-CoV-2. Serological tests performed on the owners of Cat 1 and Cat 4 showed the presence of IgG antibodies only, while the owners of Cat 2 and Cat 3 were weakly positive for IgM in addition to IgG (a weaker band compared to the control and IgG antibodies). Fecal samples were not examined due to a lack of patients meeting the inclusion criteria described in the Section 2.

The PCR tests of the samples from the four cats gave negative results for FHV, FCV, *Mycoplasma* spp. and *Chlamydia felis*.

Treatment of standalone SARS2 included Viusid 2 × 1 mL per 5 kg of body weight, R X Immunosupport 1 capsule daily, Vetomun 1 capsule daily and Lisymun 2 × 0.5 mL for up to 2.5 kg body weight and 2 × 1 mL for 2.5–5 kg kittens. The second PCR test performed 10 days after the diagnosis showed negative results. The same therapy was applied to the treatment of cases associated with co-infection. The duration of administration of these preparations was 1–3 months, depending on the severity of the disease.

**Figure 2:**
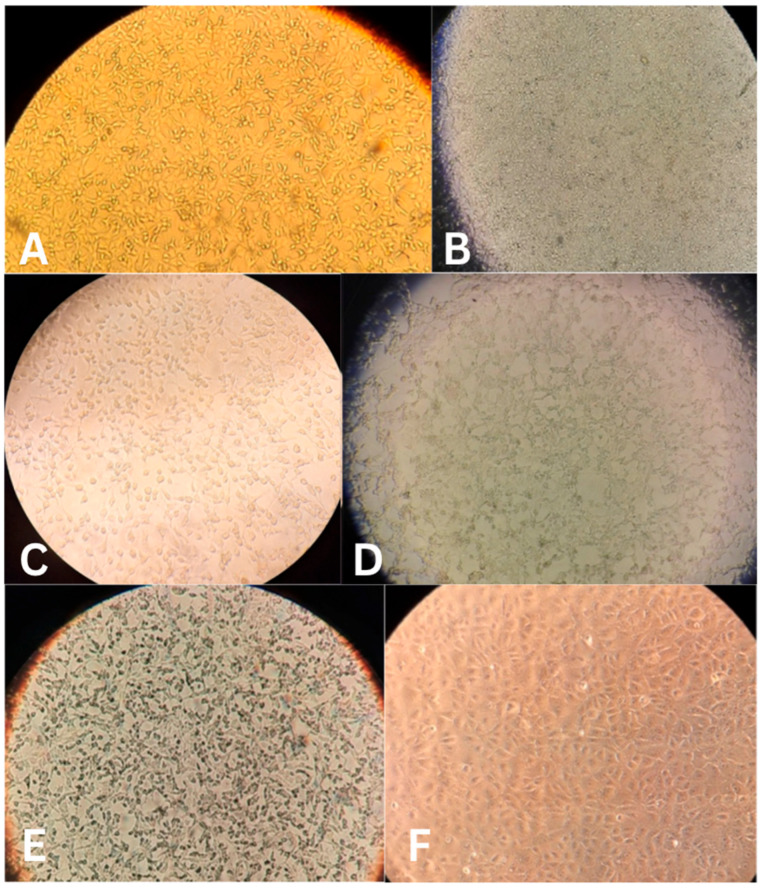
Cytopathic effect of four SARS-CoV-2 isolates from cats, a positive control from a human isolate, and cell culture control 48 h after infection. (**A**) Cat isolate 1; (**B**) Cat isolate 2; (**C**) Cat isolate 3; (**D**) Cat isolate 4; (**E**) Control isolate; (**F**) cell culture (negative) control.

Propagation of the four samples on the VERO E6 cell line resulted in characteristic CPE (Figure 2).

The viral isolates showed the following titers: Isolate 1–10^4.67^ TCID_50_/0.2 mL; Isolate 2–10^4.5^ TCID_50_/0.2 mL; Isolate 3–10^5.5^ TCID_50_/0.2 mL; and Isolate cat 4–10^5.67^ TCID_50_/0.2 mL We conducted experiments with the isolation of the virus in permissive cell cultures using conventional virology methods in order to prove its infectivity, i.e., the ability to infect. We selected samples infected only with SARS-CoV-2 to avoid contamination of the laboratory with mycoplasmas. It should be noted that the detection of a replication-competent virus in a cell culture could indicate, but is not necessarily equivalent to, infectivity or virulence in vivo. Rather, it assesses the pathogenicity of the virus in vitro—whether and to what extent it infects and changes cells, whether it is lytic, etc.

**Figure 3:**
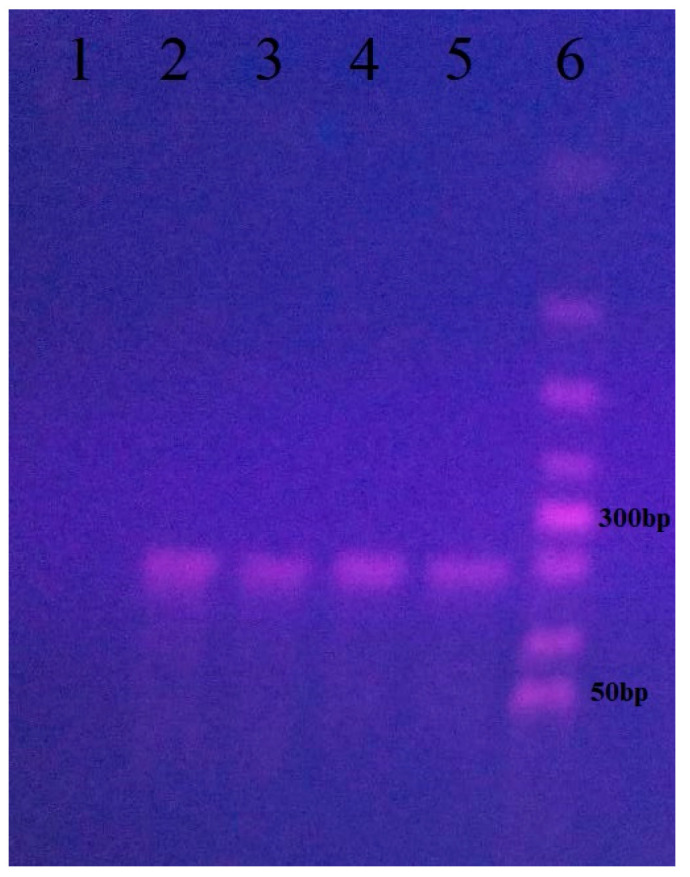
PCR analysis for confirmation of SARS-CoV-2 isolates from cell cultures from four cats positive only for the virus. (1) Negative control; (2) sample from Cat 1; (3) sample from Cat 2; (4) sample from Cat 3; (5) sample from Cat 4; (6) 50 bp DNA ladder (Bioline).

To confirm SARS-CoV-2, we performed PCR on the isolates and sequenced the resulting 212 bp products (Figure 3). Sequence alignment in MEGAX and analysis via BLAST NCBI confirmed that the isolates were SARS-CoV-2.

## 4. Discussion

The results showed detection of SARS-CoV-2 in 20.6% (21/102) of samples taken from patients with upper respiratory tract infections. In 81% of these cases, there were co-infections with FHV, *Mycoplasma* and *Chl. felis*. There was a standalone SARS-CoV-2 infection in four cats, and we obtained virus isolates from them to test for the presence of infectious SARS-CoV-2 virions and to help determine the possibility for infectious virus spreading from cats. In contrast to Thieulent et al. [30], who found 27% positive for FCV in 63 clinical samples from cats, we did not find positive results for this pathogen in our samples during the time period indicated. Several factors may explain this finding. One of these could be the timing of sampling (either after or before virus shedding). Another potentially important factor is differences in pathogen tropism and clinical presentation. In contrast to feline herpesvirus, which is strongly associated with classical upper respiratory tract signs, FCV infections are more commonly associated with oral ulcers and, in some cases, lameness. Therefore, selection of animals based primarily on respiratory clinical signs may have favored the detection of FHV-1 and other agents, such as Mycoplasma felis.

A likely reason could also be the presence of co-infecting pathogens, including SARS-CoV-2, which could also affect pathogen dynamics and the probability of detection.

Taken together, the lack of detection of FCV in this study likely reflects a combination of population-specific, clinical, and methodological factors, rather than its lack of relevance in feline respiratory disease.

A likely reason could also be the presence of co-infecting pathogens, including SARS-CoV-2, which could also affect pathogen dynamics and the probability of detection.

According to Fisher’s theory [36], the random distribution of infection between the sexes is 50:50, and this also applies to domestic cats [37].

Our results correlate with these data, with a difference of 2% (48% male cats). This is in agreement with other reports that female cats predominate slightly over males, with a difference of 4% between the sexes [25,38].

Previous serological studies of domestic and stray cats have shown no statistical relationship between sex and the risk of SARS-CoV-2 infection [8,10,28,39]. Some authors have reported higher SARS-CoV-2 prevalence in female cats in serological assays [40] and antigen tests [27], while others have reported this in males [41,42]. Our results showed that the cases of SARS-CoV-2 infection were 2-fold higher among female cats as compared to males. In our study, the difference in the number of samples between the two sexes (52% female vs. 48% male) influenced this result at a smaller scale compared to Tyson et al. [42], with a distribution of 53% male, 39% female and 8% unidentified. It is likely that behavioral factors, such as more frequent interaction with humans, may play a major role, rather than a difference in the biological susceptibility to the virus [40,42].

In cases of SARS-CoV-2 and FHV co-infection, there is probably a reactivation of the herpesvirus as a result of SARS-CoV-2 infection [26], which leads to immune system suppression in various ways. For example, there is evidence that low levels of SARS-CoV-2 N protein suppress the innate immune response by reducing RIG-I ubiquitination through interaction with TRIM25, reducing STAT1/STAT2 phosphorylation and nuclear translocation [43], and also disrupting the antiviral response by inhibiting all key stages of IFN defense [44]. It may also be the result of reactivation or recent infection, in connection with the above. Mycoplasmas cause endogenous infections, but since their virulent characteristics are not well established, it is not possible to rule out external transmission completely. Mycoplasmas can act as secondary agents when the host’s defenses weaken as a result of infection with viruses, bacteria or other causes [31]. Thus, infection with SARS-CoV-2 may ‘predispose’ the host and potentiate the development of mycoplasma infection through its negative effect on the immune system, as described above.

According to the general understanding, natural transmission of chlamydial infections occurs through objects and close contact with other affected animals and their aerosols [31]. Chlamydial infections in cats may not be associated with SARS-CoV-2, but may have an impact on the subsequent development of the infection for the reasons already mentioned.

Approximately one-third of the cats tested negative for the pathogens under investigation, potentially indicating that the clinical signs may have developed as a result of other microorganisms, such as *Pasteurella*, *Streptococcus*, *Staphylococcus*, *Pseudomonas*, *E. coli*, *Bordetella*, *Cryptococcus*, *Aspergillus* [45,46,47] or non-infectious causes such as allergies [48,49]. To investigate this, other tests such as standard microbiology and allergen tests need to be performed.

Direct detection of SARS-CoV-2 (antigen or RNA) varies in street cats from 0.5% [25] to 23.60% [27]. In our study, the domestic cats showed 20.6% positive samples, which corresponds to the possibility of contact between the domestic cat and its infected owner [26]. Hosie et al. [26], based on the prevalence of SARS-CoV-2 in humans in the UK for the period March–June 2020 (5%), estimate that 19 out of the 386 cat samples tested belong to households affected by COVID-19, with the authors detecting only one positive sample. In our study, for five of the SARS-CoV-2-positive cats, there was evidence of a respiratory infection in the owners 17–20 days prior. Based on the above, we can consider infections in cats as a manifestation of SARS-CoV-2 infection in humans and a potential indicator of a past asymptomatic or undiagnosed SARS-CoV-2 infection in the family. Based on the owners of SARS-CoV-2-positive cats, we speculate that approximately 20% of owners in this subset may have had a prior infection, whereas official data for the general population in 2023 ranged from 3 to 16% [50]. As the post-pandemic demand for testing and, accordingly, the number of people tested was low [50], we speculate that the prevalence was higher and correlated with the data for cats. The owners of all SARS-CoV-2-positive cats were clinically healthy at the time of the study. This, however, does not rule out the possibility that they were asymptomatic or oligosymptomatic carriers of the virus.

On the basis of our observations and those of other authors, we can summarize that the clinical picture of SARS-CoV-2 in cats is associated with asymptomatic [19], mild disease and symptoms such as sneezing [20], digestive signs, apathy and appetite reduction, fever, upper respiratory tract involvement [9] and serous secretions from the medial eye corner, gastrointestinal symptoms [21] or complications such as hypertrophic cardiomyopathy [22], myocarditis, pneumonia [23] and bronchial pneumonia [19]. The transmission of SARS-CoV-2 from humans to cats has been proven in owners infected with the virus: indirectly via detection of antibodies in their cats and directly via detection of the virus in the cats [8,13,28]. Viral RNA can remain detectable in cats infected with SARS-CoV-2 for up to 17 days [13]. Detection of viral nucleic acid by PCR does not indicate that the infected host is the source of infectious virus and can spread the virus. There is evidence that a SARS-CoV-2 positive PCR result does not always result in virus replication and the release of infectious virions [11]. Studies show that virus transmission is possible from cat to cat [9]. In cases of natural infection, there is evidence that antibodies are not detected until three months after infection [9], indicating that a cat can become reinfected with the virus. This, along with the prolonged spread, makes it possible for the virus to circulate in certain cat populations (large numbers over a wide area). In this respect, studies discuss the possibility of cats infecting humans and their role in the spread and epidemiology of the virus [9,13,29,40,51,52].

Viruses are not naturally adapted to laboratory cell cultures used for virus isolation. Viruses need to adapt to the respective cell culture via corresponding mutations in the viruses [53,54,55]. Isolation of a virus in laboratory conditions demonstrates that the sample contains a sufficient infectious viral load to infect susceptible hosts, thus reflecting the natural adaptation of the virus to these species. It should be noted that the detection of replication-competent virus in cell culture may indicate, but is not necessarily equivalent to, infectivity or virulence in vivo. Rather, it assesses the pathogenicity of the virus in vitro—whether and to what extent it infects and modifies cells, whether it is lytic, etc. On the other hand, the fact that the virus adapts to efficient replication in vitro allows us to speculate that transmission of the infection at the organismal level is possible. Of course, such a definitive conclusion also requires support from quantitative PCR (Ct) to determine viral load. The sequencing performed in this study targeted a relatively short genomic fragment (212 bp), (Supplementary S1) which allows reliable confirmation of SARS-CoV-2 infection. However, this fragment is insufficient for accurate variant identification or phylogenetic analysis. Therefore, lineage classification (e.g., Omicron sublineages) was not performed. Future studies using whole-genome sequencing would provide more detailed epidemiological insights.

SARS-CoV-2-positive cats may be a potential source of infection, although direct transmission was not determined in this study. This is the first component of the Gromashevsky [56] triad—the presence of a source of an infectious agent. The second component, the susceptible host, includes humans and other cats that may be exposed to the virus. The third component, the environment, is relatively constant in homes and facilitates interaction between the agent and the susceptible host, creating conditions for potential transmission of the infection to both humans and other cats.

Two of the cats from which we isolated the virus had owners who showed only IgG antibodies. These antibodies may be post-infectious or post-vaccination. The weakly positive result for IgM antibodies in two of the owners is probably due to a fading initial secondary immune response to SARS-CoV-2. The virus transmission occurred probably from the owners to the cats. It is less likely that the cats had acquired the infection in another way, such as through contaminated shoes of the owners or guests. It is also possible for the virus transmission to occur from cat to cat [9]. In such cases, the cat could transmit the virus to the owner and, in the absence of post-infectious IgA immunity on his part [57], local infection with SARS-CoV-2 could develop.

Although the infection has a mild clinical course in cats, preparations such as Viusid, R X Immunosupport, Vetomun and Lisymun can support the immune response owing to their antioxidant and immunomodulatory (stimulating) properties. The second PCR, 10 days after the infection onset, did not detect the virus, and there were no clinical signs. This allows us to speculate that the therapy may have a potential effect on the elimination of the virus and on the shedding period. However, the present study does not allow us to establish a definitive causal relationship.

It is important for owners to limit their cats’ contact with shoes and clothing worn outdoors and to wash their hands before touching their pets. The study of domestic cats in the SARS-CoV-2 post-pandemic period is important for the differential diagnosis of the causal agents of conjunctivitis and upper respiratory tract diseases, as well as for understanding the movement and spread of the virus in the environment close to humans.

The study has some limitations. The ELISA method used in our study does not distinguish between different classes of antibodies and therefore does not provide information about current or past infection. In this regard, the method can find application as an additional test in cases of suspected SARS-CoV-2 infection shortly after infection, as antibodies are not detected at 3 months [9]. The owners did not give permission for taking follow-up blood samples from their cats (20 days later) to test for the presence of antibodies against SARS-CoV-2, to demonstrate seroconversion, or for subsequent samples to study the dynamics of the immune response. Also, fecal samples were not tested due to the lack of patients that met the eligibility criteria. Therefore, we were unable to test for shedding through feces and to assess the role of such shedding in environmental contamination and virus spread. Lastly, variant identification was beyond the scope of the present study. The sequencing performed targeted a short fragment (212 bp), which is sufficient for confirmation of SARS-CoV-2 but does not allow reliable lineage classification. Due to the short length of the sequenced fragment, phylogenetic analysis would not provide reliable results. Therefore, we limited our analysis to BLAST confirmation.

## 5. Conclusions

This study showed that domestic cats can shed infectious SARS-CoV-2, even with minimal clinical signs, and indoor owners were the possible source of infection. The results highlight the role of cats as potential sources of infection and contribute to the understanding of the movement and spread of SARS-CoV-2 in domestic environments. The reported observations also contribute to the differential diagnosis of respiratory and ophthalmic diseases in cats. The results from immunotherapy indicated a potential effect on the immune response in post-pandemic SARS-CoV-2 infection. These findings may suggest a potential supportive role of immunomodulatory therapy; however, no causal relationship can be established within the present study design.

The main contributions of this study are its demonstration of infectious SARS-CoV-2 in naturally infected cats, its offering of post-pandemic data (2023), and its integration of molecular, serological and co-infection diagnostics.

This single-center study demonstrates that infectious SARS-CoV-2 can be isolated from symptomatic domestic cats during the post-pandemic period. Most PCR-positive cats had co-infections with other respiratory pathogens. In the study, we report our findings of infection and coinfection of domestic cats in post-pandemic samples, recognizing that large-scale epidemiological studies are needed to address the fundamental scientific question of whether cats can be a reservoir of SARS-CoV-2. The clinical signs were mild. Further controlled and longitudinal studies are required to determine the epidemiological significance and clinical impact of feline SARS-CoV-2 infection.

## Figures and Tables

**Table 1:** (**A**). Descriptive statistics of age in the studied cats. (**B**). Sex distribution of the studied cats.

**(A)**		
**Parameter**	**Value**	
Sample size (*n*)	71	
Minimum (years)	0.17	
Maximum (years)	18.00	
Mean ± SD (years)	4.36 ± 4.16	
95% CI (mean)	3.37–5.34	
Median (years)	3.00	
95% CI (median)	2.00–5.00	
Variance	17.34	
Standard error (SEM)	0.49	
Coefficient of variation (%)	95.61	
Skewness	1.02 (*p* = 0.0011)	
Kurtosis	0.41 (*p* = 0.3761)	
Shapiro–Wilk test	W = 0.8715, *p* < 0.0001	
Distribution	Non-normal	
**(B)**		
**Sex**	**Number (** ***n*** **)**	**Percentage (%)**
Female	53	52
Male	49	48
Total	102	100

Data show median values instead of arithmetic means due to uneven age distribution.

**Table 2:** (**A**). Types of co-infections among SARS-CoV-2–positive cats. (**B**). Statistical analysis.

**(A)**		
**Infection Type**	**Number (** ***n*** **)**	**Percent (%)**
SARS-CoV-2 + *Mycoplasma* spp.	8	38.1
SARS-CoV-2 + *Mycoplasma* spp. + *C. felis*	2	9.5
SARS-CoV-2 + *Mycoplasma* spp. + FHV	2	9.5
SARS-CoV-2 + *Mycoplasma* spp. + FHV + *C. felis*	5	23.8
SARS-CoV-2 only	4	19.0
Total	21	100
**(B)**		
**Statistic**	**Value**	
Chi-squared (χ^2^)	5.905	
Degrees of freedom (DF)	4	
Significance level	*p* = 0.2064	

**Table 3:** Identified co-infections without SARS-CoV-2 involvement in 102 clinical samples from cats in 2023.

**Type of Co-Infection**	**Number (** ***n*** **)**	**Percent (%)**
*C. felis* + *Mycoplasma* spp.	3	18.75
FHV + *C. felis*	4	25.00
FHV + *C. felis* + *Mycoplasma* spp.	3	18.75
FHV + *Mycoplasma* spp.	6	37.50
Total	16	100.00

## Data Availability

The original contributions presented in this study are included in the article/Supplementary Material. Further inquiries can be directed to the corresponding author(s).

## Supplementary Materials

The following supporting information can be downloaded at https://www.mdpi.com/article/10.3390/vetsci13040374/s1: Supplementary S1. Partial sequences of the N protein of cultured SARS-CoV-2 from cats and confirmation in BLAST, NCBI.

## Abbreviations

The following abbreviations are used in this manuscript:

FHV	*Feline herpesvirus*
FCV	*Feline calicivirus*
PCR	Polymerase chain reaction
CPE	Cytopathological effect
URT	Upper respiratory tract
RNA	Ribonucleic acid
DNA	Deoxyribonucleic acid
ELISA	Enzyme-Linked Immunosorbent Assay
TRIM25	Tripartite Motif Containing 25
