# Supplementary material for: Prevalence, Clinical Signs, Diagnosis and Treatment of Post-Pandemic SARS-CoV-2 Infection in Cats in 2023: Co-Infection with FHV, FCV, Mycoplasma spp. and Chlamydia felis—A Single-Center Study in Bulgaria
Source: Vet Sci. 2026 Apr 13;13(4):374. doi: 10.3390/vetsci13040374 (PMC13119600; doi:10.3390/vetsci13040374)
Supplement: Supplementary file 1 [file vetsci-13-00374-s001.zip › vetsci-4147273-supplementary.pdf]

The quality of the sequences allowed us to separate the following fragments for identification in NCBI (without background and without the need for additional processing; during cultivation the virus changes and therefore the "clean" regions become shorter. This is the subject of another study of ours).

Supplementary S1. Partial sequences of the N protein of cultured SARS-CoV-2 from cats and confirmation in BLAST, NCBI.

CTCAACATGGCAAGGAAGACCTTAAATTCCCTCGAGGACAAGGCGTTCCAATTAACACCAATAGCAGTCCAGATG ACCAAATTGGCTACTACCGAAGAGCTACCAGACGAATTCGTGGTGGTGACGGTAAAATGAAAGAT

select all 100 sequences selected GenBank Graphics Distance tree of results MSA Viewer

	Description	Scientific Name	Max Score	Total Score	Query Cover	E value	Per. Ident	Acc. Len	Accession
☑	Severe acute respiratory syndrome coronavirus 2 isolate SARS-CoV-2/human/USA/CO-CDC-FG-266366/2022 ...	Severe acute res...	259	259	100%	7e-65	100.00%	29821	OM700324.1
☑	Severe acute respiratory syndrome coronavirus 2 isolate SARS-CoV-2/human/USA/CO-CDPHE-2103132151/20...	Severe acute res...	259	259	100%	7e-65	100.00%	29746	ON356157.1
☑	Severe acute respiratory syndrome coronavirus 2 isolate SARS-CoV-2/human/USA/VA-CDC-QDX47920148/202...	Severe acute res...	259	259	100%	7e-65	100.00%	29721	OQ705059.1
☑	Severe acute respiratory syndrome coronavirus 2 isolate SARS-CoV-2/human/USA/UT-UPHL-210701618455/20...	Severe acute res...	259	259	100%	7e-65	100.00%	29782	MZ914262.1
☑	Severe acute respiratory syndrome coronavirus 2 isolate SARS-CoV-2/human/USA/MA-CDC-LC0492082/2022, ...	Severe acute res...	259	259	100%	7e-65	100.00%	29717	OM496802.1
☑	Severe acute respiratory syndrome coronavirus 2 genome assembly, chromosome: 1	Severe acute res...	259	259	100%	7e-65	100.00%	29850	OW365772.1
☑	Severe acute respiratory syndrome coronavirus 2 isolate SARS-CoV-2/human/USA/NV-CDC-2-7062996/2023 O...	Severe acute res...	259	259	100%	7e-65	100.00%	29793	OR099031.1
☑	Severe acute respiratory syndrome coronavirus 2 isolate SARS-CoV-2/human/USA/OR-CDC-LC0470279/2022 ...	Severe acute res...	259	259	100%	7e-65	100.00%	29502	OM367756.1
☑	Severe acute respiratory syndrome coronavirus 2 genome assembly, chromosome: 1	Severe acute res...	259	259	100%	7e-65	100.00%	29885	OV915106.1
☑	Severe acute respiratory syndrome coronavirus 2 genome assembly, chromosome: 1	Severe acute res...	259	259	100%	7e-65	100.00%	29864	OW724371.1
☑	Severe acute respiratory syndrome coronavirus 2 genome assembly, chromosome: 1	Severe acute res...	259	259	100%	7e-65	100.00%	29850	OW388100.1
☑	Severe acute respiratory syndrome coronavirus 2 isolate SARS-CoV-2/human/USA/NJ-CDC-QDX26115779/202...	Severe acute res...	259	259	100%	7e-65	100.00%	29763	MZ530467.1
☑	Severe acute respiratory syndrome coronavirus 2 isolate SARS-CoV-2/human/USA/CO-CDPHE-2103011786/20...	Severe acute res...	259	259	100%	7e-65	100.00%	29481	ON104024.1
☑	Severe acute respiratory syndrome coronavirus 2 isolate SARS-CoV-2/human/USA/TX-CDC-FG-255534/2022, c...	Severe acute res...	259	259	100%	7e-65	100.00%	29830	OM573952.1
☑	Severe acute respiratory syndrome coronavirus 2 isolate SARS-CoV-2/human/IND/Sample095/2022 ORF1ab pol...	Severe acute res...	259	259	100%	7e-65	100.00%	29706	OQ587527.1

[illegible] (ORF1ab), ORF1a polyprotein (ORF1ab), surface glycoprotein (S), ORF3a protein (ORF3a), envelope protein (E), membrane glycoprotein (M), ORF6 protein (ORF6), ORF7a protein (ORF7a), and ORF7b (ORF7b) genes, complete cds; ORF8 gene, complete sequence; and nucleocapsid phosphoprotein (N) and ORF10 protein (ORF10) genes, complete cds

Sequence ID: OQ705059.1 Length: 29721 Number of Matches: 1

Range 1: 28363 to 28502 GenBank Graphics ▼ Next Match ▲ Previous Match

Score	Expect	Identities	Gaps	Strand
259 bits(140)	7e-65	140/140(100%)	0/140(0%)	Plus/Plus

```
Query  1      CTCAACATGGCAAGGAAGACCTTAAATTCCCTCGAGGACAAGGCGTTCCAATTAACACCA  60
              ||||||||||||||||||||||||||||||||||||||||||||||||||||||||||||
Sbjct  28363  CTCAACATGGCAAGGAAGACCTTAAATTCCCTCGAGGACAAGGCGTTCCAATTAACACCA  28422

Query  61     ATAGCAGTCCAGATGACCAAATTGGCTACTACCGAAGAGCTACCAGACGAATTCGTGGTG  120
              ||||||||||||||||||||||||||||||||||||||||||||||||||||||||||||
Sbjct  28423  ATAGCAGTCCAGATGACCAAATTGGCTACTACCGAAGAGCTACCAGACGAATTCGTGGTG  28482

Query  121    GTGACGGTAAAATGAAAGAT  140
              ||||||||||||||||||||
Sbjct  28483  GTGACGGTAAAATGAAAGAT  28502
```

Download ⌄ GenBank Graphics ▼ Next ▲ Previous ◄Descriptions

Severe acute respiratory syndrome coronavirus 2 isolate SARS-CoV-2/human/USA/UT-UPHL-210701618455/2021, complete genome

Sequence ID: MZ914262.1 Length: 29782 Number of Matches: 1

Range 1: 28389 to 28528 GenBank Graphics ▼ Next Match ▲ Previous Match

Score	Expect	Identities	Gaps	Strand
259 bits(140)	7e-65	140/140(100%)	0/140(0%)	Plus/Plus

```
Query  1      CTCAACATGGCAAGGAAGACCTTAAATTCCCTCGAGGACAAGGCGTTCCAATTAACACCA  60
              ||||||||||||||||||||||||||||||||||||||||||||||||||||||||||||
Sbjct  28389  CTCAACATGGCAAGGAAGACCTTAAATTCCCTCGAGGACAAGGCGTTCCAATTAACACCA  28448

Query  61     ATAGCAGTCCAGATGACCAAATTGGCTACTACCGAAGAGCTACCAGACGAATTCGTGGTG  120
              ||||||||||||||||||||||||||||||||||||||||||||||||||||||||||||
Sbjct  28449  ATAGCAGTCCAGATGACCAAATTGGCTACTACCGAAGAGCTACCAGACGAATTCGTGGTG  28508

Query  121    GTGACGGTAAAATGAAAGAT  140
              ||||||||||||||||||||
Sbjct  28509  GTGACGGTAAAATGAAAGAT  28528
```

AGCAGTCCAGATGACCAAATTGGCTACTACCGAAGAGCTACCAGACGAATTCGTGGTGGTGACGGTAAAATGAAA GATCTCAGTCCAAGATGGTATTT

Descriptions | Graphic Summary | Alignments | Taxonomy

Sequences producing significant alignments

Download | Select columns | Show 100

select all *100 sequences selected*

GenBank Graphics Distance tree of results MSA Viewer

Description	Scientific Name	Max Score	Total Score	Query Cover	E value	Per. Ident	Acc. Len	Accession
Severe acute respiratory syndrome coronavirus 2 isolate SARS-CoV-2/human/USA/CO-CDC-FG-266366/2022 ...	Severe acute res...	182	182	100%	1e-41	100.00%	29821	OM700324.1
Severe acute respiratory syndrome coronavirus 2 isolate SARS-CoV-2/human/USA/CO-CDPHE-2103132151/20...	Severe acute res...	182	182	100%	1e-41	100.00%	29746	ON356157.1
Severe acute respiratory syndrome coronavirus 2 genome assembly, chromosome: 1	Severe acute res...	182	182	100%	1e-41	100.00%	29890	OV056950.1
Severe acute respiratory syndrome coronavirus 2 isolate SARS-CoV-2/human/USA/VA-CDC-QDX47920148/202...	Severe acute res...	182	182	100%	1e-41	100.00%	29721	OQ705059.1
Severe acute respiratory syndrome coronavirus 2 genome assembly, chromosome: 1	Severe acute res...	182	182	100%	1e-41	100.00%	29823	OV632015.1
Severe acute respiratory syndrome coronavirus 2 isolate SARS-CoV-2/human/USA/UT-UPHL-210701618455/20...	Severe acute res...	182	182	100%	1e-41	100.00%	29782	MZ914262.1
Severe acute respiratory syndrome coronavirus 2 isolate SARS-CoV-2/human/USA/MA-CDC-STM-HSHAY5GK3...	Severe acute res...	182	182	100%	1e-41	100.00%	29888	OL805771.1
Severe acute respiratory syndrome coronavirus 2 isolate SARS-CoV-2/human/USA/MA-CDC-LC0492082/2022, ...	Severe acute res...	182	182	100%	1e-41	100.00%	29717	OM496802.1
Severe acute respiratory syndrome coronavirus 2 isolate SARS-CoV-2/human/USA/MO-CDC-LC0416318/2021, ...	Severe acute res...	182	182	100%	1e-41	100.00%	29661	OL882915.1
Severe acute respiratory syndrome coronavirus 2 genome assembly, chromosome: 1	Severe acute res...	182	182	100%	1e-41	100.00%	29850	OW365772.1
Severe acute respiratory syndrome coronavirus 2 isolate SARS-CoV-2/human/USA/NV-CDC-2-7062996/2023 O...	Severe acute res...	182	182	100%	1e-41	100.00%	29793	OR099031.1
Severe acute respiratory syndrome coronavirus 2 isolate SARS-CoV-2/human/USA/OR-CDC-LC0470279/2022 ...	Severe acute res...	182	182	100%	1e-41	100.00%	29502	OM367756.1
Severe acute respiratory syndrome coronavirus 2 genome assembly, chromosome: 1	Severe acute res...	182	182	100%	1e-41	100.00%	29890	OV056364.1
Severe acute respiratory syndrome coronavirus 2 genome assembly, chromosome: 1	Severe acute res...	182	182	100%	1e-41	100.00%	29859	OV497878.1
Severe acute respiratory syndrome coronavirus 2 genome assembly, chromosome: 1	Severe acute res...	182	182	100%	1e-41	100.00%	29890	OV256208.1
Severe acute respiratory syndrome coronavirus 2 genome assembly, chromosome: 1	Severe acute res...	182	182	100%	1e-41	100.00%	29885	OV915106.1
Severe acute respiratory syndrome coronavirus 2 genome assembly, chromosome: 1	Severe acute res...	182	182	100%	1e-41	100.00%	29864	OW724371.1
Severe acute respiratory syndrome coronavirus 2 isolate SARS-CoV-2/human/USA/NJ-PHEL-V21008465/2021 ...	Severe acute res...	182	182	100%	1e-41	100.00%	29785	OK002014.1
Severe acute respiratory syndrome coronavirus 2 genome assembly, complete genome: monopartite	Severe acute res...	182	182	100%	1e-41	100.00%	29890	OY407283.1

Download GenBank Graphics ▼ Next ▲ Previous ◀ Descriptions

Severe acute respiratory syndrome coronavirus 2 isolate SARS-CoV-2/human/USA/CO-CDPHE-2103132151/2021 ORF1ab polyprotein (ORF1ab), ORF1a polyprotein (ORF1ab), surface glycoprotein (S), ORF3a protein (ORF3a), envelope protein (E), membrane glycoprotein (M), ORF6 protein (ORF6), and ORF7a protein (ORF7a) genes, complete cds; ORF7b (ORF7b) and ORF8 protein (ORF8) genes, partial cds; and nucleocapsid phosphoprotein (N) and ORF10 protein (ORF10) genes, complete cds

Sequence ID: ON356157.1 **Length:** 29746 **Number of Matches:** 1

Range 1: 28415 to 28512 GenBank Graphics ▼ Next Match ▲ Previous Match

Score	Expect	Identities	Gaps	Strand
182 bits(98)	1e-41	98/98(100%)	0/98(0%)	Plus/Plus

```
Query  1      AGCAGTCCAGATGACCAAATTGGCTACTACCGAAGAGCTACCAGACGAATTCGTGGTGGT  60
              ||||||||||||||||||||||||||||||||||||||||||||||||||||||||||||
Sbjct  28415  AGCAGTCCAGATGACCAAATTGGCTACTACCGAAGAGCTACCAGACGAATTCGTGGTGGT  28474

Query  61     GACGGTAAAATGAAAGATCTCAGTCCAAGATGGTATTT  98
              ||||||||||||||||||||||||||||||||||||||
Sbjct  28475  GACGGTAAAATGAAAGATCTCAGTCCAAGATGGTATTT  28512
```

Download GenBank Graphics ▼ Next ▲ Previous ◀ Descriptions

Severe acute respiratory syndrome coronavirus 2 genome assembly, chromosome: 1

Sequence ID: OV056950.1 **Length:** 29890 **Number of Matches:** 1

Range 1: 28492 to 28589 GenBank Graphics ▼ Next Match ▲ Previous Match

Score	Expect	Identities	Gaps	Strand
182 bits(98)	1e-41	98/98(100%)	0/98(0%)	Plus/Plus

```
Query  1      AGCAGTCCAGATGACCAAATTGGCTACTACCGAAGAGCTACCAGACGAATTCGTGGTGGT  60
              ||||||||||||||||||||||||||||||||||||||||||||||||||||||||||||
Sbjct  28492  AGCAGTCCAGATGACCAAATTGGCTACTACCGAAGAGCTACCAGACGAATTCGTGGTGGT  28551

Query  61     GACGGTAAAATGAAAGATCTCAGTCCAAGATGGTATTT  98
              ||||||||||||||||||||||||||||||||||||||
Sbjct  28552  GACGGTAAAATGAAAGATCTCAGTCCAAGATGGTATTT  28589
```

TCCAATTAACACCAATAGCAGTCCAGATGACCAAATTGGCTACTACCGAAGAGCTACCAGACGAATTCGTGGTGGT
GACGGTAAAATGAAAGATCTCAGTCCAAGATGGTATTTCTACTACCTAGGAACTGGGCCAGAAGCT

Descriptions | Graphic Summary | Alignments | Taxonomy

Sequences producing significant alignments Download Select columns Show 100

select all *100 sequences selected* GenBank Graphics Distance tree of results MSA Viewer

	Description	Scientific Name	Max Score	Total Score	Query Cover	E value	Per. Ident	Acc. Len	Accession
☑	Severe acute respiratory syndrome coronavirus 2 isolate SARS-CoV-2/human/USA/CO-CDC-FG-266366/2022 ...	Severe acute res...	263	263	100%	6e-66	100.00%	29821	OM700324.1
☑	Severe acute respiratory syndrome coronavirus 2 isolate SARS-CoV-2/human/USA/CO-CDPHE-2103132151/20...	Severe acute res...	263	263	100%	6e-66	100.00%	29746	ON356157.1
☑	Severe acute respiratory syndrome coronavirus 2 genome assembly, chromosome: 1	Severe acute res...	263	263	100%	6e-66	100.00%	29890	OV056950.1
☑	Severe acute respiratory syndrome coronavirus 2 isolate SARS-CoV-2/human/USA/VA-CDC-QDX47920148/202...	Severe acute res...	263	263	100%	6e-66	100.00%	29721	OQ705059.1
☑	Severe acute respiratory syndrome coronavirus 2 genome assembly, chromosome: 1	Severe acute res...	263	263	100%	6e-66	100.00%	29823	OV632015.1
☑	Severe acute respiratory syndrome coronavirus 2 isolate SARS-CoV-2/human/USA/UT-UPHL-210701618455/20...	Severe acute res...	263	263	100%	6e-66	100.00%	29782	MZ914262.1
☑	Severe acute respiratory syndrome coronavirus 2 isolate SARS-CoV-2/human/USA/MA-CDC-STM-HSHAY5GK3...	Severe acute res...	263	263	100%	6e-66	100.00%	29888	OL805771.1
☑	Severe acute respiratory syndrome coronavirus 2 isolate SARS-CoV-2/human/USA/MA-CDC-LC0492082/2022, ...	Severe acute res...	263	263	100%	6e-66	100.00%	29717	OM496802.1
☑	Severe acute respiratory syndrome coronavirus 2 isolate SARS-CoV-2/human/USA/MO-CDC-LC0416318/2021, ...	Severe acute res...	263	263	100%	6e-66	100.00%	29661	OL882915.1
☑	Severe acute respiratory syndrome coronavirus 2 genome assembly, chromosome: 1	Severe acute res...	263	263	100%	6e-66	100.00%	29850	OW365772.1
☑	Severe acute respiratory syndrome coronavirus 2 isolate SARS-CoV-2/human/USA/NV-CDC-2-7062996/2023 O...	Severe acute res...	263	263	100%	6e-66	100.00%	29793	OR099031.1
☑	Severe acute respiratory syndrome coronavirus 2 isolate SARS-CoV-2/human/USA/OR-CDC-LC0470279/2022 ...	Severe acute res...	263	263	100%	6e-66	100.00%	29502	OM367756.1
☑	Severe acute respiratory syndrome coronavirus 2 genome assembly, chromosome: 1	Severe acute res...	263	263	100%	6e-66	100.00%	29890	OV056364.1
☑	Severe acute respiratory syndrome coronavirus 2 genome assembly, chromosome: 1	Severe acute res...	263	263	100%	6e-66	100.00%	29859	OV497878.1
☑	Severe acute respiratory syndrome coronavirus 2 genome assembly, chromosome: 1	Severe acute res...	263	263	100%	6e-66	100.00%	29890	OV256208.1
☑	Severe acute respiratory syndrome coronavirus 2 genome assembly, chromosome: 1	Severe acute res...	263	263	100%	6e-66	100.00%	29885	OV915106.1
☑	Severe acute respiratory syndrome coronavirus 2 genome assembly, chromosome: 1	Severe acute res...	263	263	100%	6e-66	100.00%	29864	OW724371.1
☑	Severe acute respiratory syndrome coronavirus 2 isolate SARS-CoV-2/human/USA/NJ-PHEL-V21008465/2021 ...	Severe acute res...	263	263	100%	6e-66	100.00%	29785	OK002014.1
☑	Severe acute respiratory syndrome coronavirus 2 genome assembly, complete genome: monopartite	Severe acute res...	263	263	100%	6e-66	100.00%	29890	OY407283.1

Severe acute respiratory syndrome coronavirus 2 isolate SARS-CoV-2/human/USA/CO-CDC-FG-266366/2022 ORF1ab polyprotein (ORF1ab), ORF1a polyprotein (ORF1ab), surface glycoprotein (S), and ORF3a protein (ORF3a) genes, complete cds; envelope protein (E) gene, partial cds; and membrane glycoprotein (M), ORF6 protein (ORF6), ORF7a protein (ORF7a), ORF7b (ORF7b), ORF8 protein (ORF8), nucleocapsid phosphoprotein (N), and ORF10 protein (ORF10) genes, complete cds

Sequence ID: OM700324.1 **Length:** 29821 **Number of Matches:** 1

Range 1: 28448 to 28589 GenBank Graphics ▼ Next Match ▲ Previous Match

Score	Expect	Identities	Gaps	Strand
263 bits(142)	6e-66	142/142(100%)	0/142(0%)	Plus/Plus

```
Query  1      TCCAATTAACACCAATAGCAGTCCAGATGACCAAATTGGCTACTACCGAAGAGCTACCAG  60
              ||||||||||||||||||||||||||||||||||||||||||||||||||||||||||||
Sbjct  28448  TCCAATTAACACCAATAGCAGTCCAGATGACCAAATTGGCTACTACCGAAGAGCTACCAG  28507

Query  61     ACGAATTCGTGGTGGTGACGGTAAAATGAAAGATCTCAGTCCAAGATGGTATTTCTACTA  120
              ||||||||||||||||||||||||||||||||||||||||||||||||||||||||||||
Sbjct  28508  ACGAATTCGTGGTGGTGACGGTAAAATGAAAGATCTCAGTCCAAGATGGTATTTCTACTA  28567

Query  121    CCTAGGAACTGGGCCAGAAGCT  142
              ||||||||||||||||||||||
Sbjct  28568  CCTAGGAACTGGGCCAGAAGCT  28589
```

Download GenBank Graphics ▼ Next ▲ Previous ◀ Descriptions

Severe acute respiratory syndrome coronavirus 2 isolate SARS-CoV-2/human/USA/CO-CDPHE-2103132151/2021 ORF1ab polyprotein (ORF1ab), ORF1a polyprotein (ORF1ab), surface glycoprotein (S), ORF3a protein (ORF3a), envelope protein (E), membrane glycoprotein (M), ORF6 protein (ORF6), and ORF7a protein (ORF7a) genes, complete cds; ORF7b (ORF7b) and ORF8 protein (ORF8) genes, partial cds; and nucleocapsid phosphoprotein (N) and ORF10 protein (ORF10) genes, complete cds

Sequence ID: ON356157.1 **Length:** 29746 **Number of Matches:** 1

Range 1: 28399 to 28540 GenBank Graphics ▼ Next Match ▲ Previous Match

Score	Expect	Identities	Gaps	Strand
263 bits(142)	6e-66	142/142(100%)	0/142(0%)	Plus/Plus

```
Query  1      TCCAATTAACACCAATAGCAGTCCAGATGACCAAATTGGCTACTACCGAAGAGCTACCAG  60
              ||||||||||||||||||||||||||||||||||||||||||||||||||||||||||||
Sbjct  28399  [illegible]  28458
```
